# Dimerized Power: The Antimicrobial and Antiviral Promise of Biflavonoids

**DOI:** 10.3390/biom16010024

**Published:** 2025-12-23

**Authors:** Hatice Duman, Sercan Karav, Anita Šalić, Dunja Šamec

**Affiliations:** 1 Department of Molecular Biology and Genetics, Çanakkale Onsekiz Mart University, Çanakkale 17100, Türkiye; hatice.duman@comu.edu.tr (H.D.); sercankarav@comu.edu.tr (S.K.); 2 University of Zagreb Faculty of Chemical Engineering and Technology, Trg Marka Marulića 19, 10000 Zagreb, Croatia; asalic@fkit.unizg.hr; 3 Department of Food Technology, University North, Trg dr. Žarka Dolinara 1, 48000 Koprivnica, Croatia

**Keywords:** biflavonoids, flavonoid dimers, natural products, antimicrobial activity, antibacterial, antifungal, antiviral, structure–activity relationship

## Abstract

Biflavonoids, a unique class of naturally occurring polyphenolic compounds formed by the dimerization of flavonoid units, have attracted increasing scientific interest due to their diverse biological activities and structural complexity. This review provides a comprehensive overview of their natural occurrence, synthetic strategies, and antimicrobial properties, with a particular focus on their antibacterial, antifungal, and antiviral activities. Comparative analyses indicate that biflavonoids often display enhanced or distinct biological effects compared to their monomeric flavonoid counterparts, suggesting that dimerization plays a crucial role in modulating bioactivity. Overall, biflavonoids represent an underexplored yet highly promising group of natural compounds with significant potential for the development of novel antimicrobial and antiviral agents. Continued interdisciplinary research integrating natural product chemistry, pharmacology, and computational modeling will be essential to fully realize their therapeutic potential.

## 1. Introduction

Flavonoids have long been among the most extensively studied groups of plant specialized metabolites, with dietary intake linked to a wide range of health benefits, including protective effects against cardiovascular and metabolic diseases [1], cancer [2], mental disorders [3], neurodegenerative pathologies [4], and other conditions. However, flavonoids represent a large and chemically diverse class of metabolites, and their biological activities can vary significantly depending on structural features [5]. For instance, the 2,3-double bond, 4-keto group, 3′,4′-catechol structure, and 3-hydroxyl group are critical for antioxidant activity, whereas 3-O-methylquercetin shows low cytotoxicity in cell proliferation assays [6].

In food, flavonoids occur either in their free (aglycone) form or as conjugates, though in plants they are most commonly present as glycosides. These glycosides are formed by bonding flavonoid aglycones with sugar moieties, typically at the C3 or C7 positions [7]. Common sugars include *L*-rhamnose, *D*-glucose, glucose-rhamnose, galactose, and arabinose [7]. Glycosylation influences the biological activity of flavonoids by improving water solubility, reducing toxicity, and enhancing selectivity of action [8]. Aglycones typically contain hydroxyl groups at C3, C5, C7, C3′, C4′, and C5′, which can undergo further modifications such as methylation, acetylation, or sulfation [7]. Prenylation, which usually occurs on the aromatic rings but can also involve O-prenylation, is another important modification that enhances biological activity [7]. Prenyl groups introduced at different positions on the flavonoid backbone can increase anti-inflammatory effects, enzyme inhibition, and estrogenic activity [9]. Structural variations, such as hydroxylation at C5, C7, C3′, and C4′ or prenylation at C6, generally enhance antibacterial activity, whereas methoxylation at C3′ and C5 reduces it [5].

Beyond monomeric forms, flavonoids also occur as dimers, known as biflavonoids. To date, nearly 600 biflavonoid structures have been reported across angiosperms, bryophytes, ferns, and gymnosperms, often in medicinal plants [10]. They can also be found in foods, where they should be distinguished from proanthocyanidins—polymers formed from flavan-3-ol monomers such as catechins and epicatechins [11]. Biflavonoids consist of flavone or flavanone units linked via C–C or C–O–C interflavonoid bonds, involving the A-, B-, or C-rings of the monomers [10]. Importantly, biflavonoids often display biological activities distinct from their monomeric subunits. Recent studies associate them with antimicrobial (particularly antiviral), neuroprotective, and anticancer properties [12]. Some researchers suggest that dimerization could serve as a natural or synthetic strategy to enhance biological potential, although the exact impact of dimerization on specific activities, such as antimicrobial effects, remains unclear.

Dimerized flavonoids have recently attracted increasing interest in the field of antimicrobial drug development due to their unique structural features and distinct biological activities. In recent years, progress has been made in understanding their therapeutic potential. Therefore, in this review, we summarize the current information on the antimicrobial properties of flavonoid dimers, with particular emphasis on their chemical diversity, biological significance, and possible applications in combating microbial resistance.

## 2. Biflavonoids

### 2.1. Chemistry and Natural Occurrence

**Figure 1:**
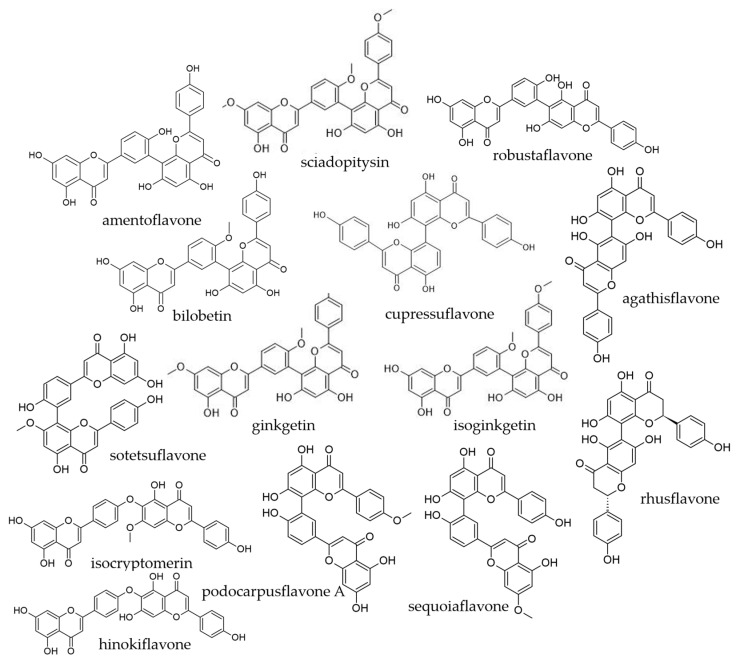
Chemical structures of representative biflavonoids.

The first biflavonoid, ginkgetin, was isolated from yellow *Ginkgo biloba* leaves in 1920. Its structure was later confirmed as a biflavonoid, and since then, numerous additional biflavonoids have been identified. According to a comprehensive review by He et al. [10], 592 biflavonoids have been structurally elucidated to date. Their structures range from relatively simple dimers, in which two flavonoid monomers are directly linked, to more complex arrangements involving different types of interflavonoid bonds. Biflavonoids can differ in their mode of linkage: in some molecules, the two flavonoid units are joined by a C–C bond (amentoflavone and derivatives, robustaflavone, etc.), while in others, they are connected through a C–O–C bridge (for example, hinokiflavone, isocryptomerin). Examples of biflavonoid structures are shown in Figure 1.

Monomeric flavonoids are typically categorized into several subclasses, including flavans, flavones, anthocyanidins, isoflavans, isoflavones, neoflavans, chalcones, aurones, and xanthones. By definition, biflavonoids consist of two such monomers linked together through various bonding patterns. This structural diversity creates the potential for an enormous number of distinct biflavonoid molecules. However, compared to their monomeric counterparts, biflavonoids have received far less scientific attention, suggesting that many naturally occurring structures likely remain undiscovered.

Biflavonoids have been reported in angiosperms, bryophytes, ferns, and gymnosperms [10], although their precise distribution across plant taxa is not yet well characterized. Much of the available literature reports the occurrence of specific biflavonoids in particular plant species, often based on sporadic findings rather than systematic surveys. For example, a recent review summarizing the occurrence of only one type of biflavonoids, the 3′,8″-biflavones, reported their presence in more than 144 species belonging to both Pteridophyta and Angiosperms [13]. Some plant species, such as *G. biloba*, accumulate only a single type of biflavonoid, in this case, the 3′,8″-biflavones [14]. In contrast, other plants, such as those from the genus *Juniperus*, produce several different biflavone types, including amentoflavone and bilobetin (both 3′,8″-type), cupressuflavone (8,8″-type), and hinokiflavone (4′,6″-type) [15]. Other examples are available in Table 1. Biflavones are most commonly found in plants in their aglycone form; however, in certain species such as whisk fern *(Psilotum nudum)* [16] or *Garcinia madruno* L. [17], glycosylated derivatives have also been reported. According to several studies on different plant species, their accumulation is tissue-specific and occurs mainly in plant parts that are in direct contact with the environment [14,16]. In food-related plants, biflavonoids have been reported in ginkgo (*G. biloba* L.), mangosteen (*Garcinia mangostana* L.), and onion (*Allium cepa* L.). For instance, ginkgo leaves contain at least 13 different biflavonoids, while species of *Garcinia* harbor around 30 biflavonoids [18]. However, due to nomenclature inconsistencies in earlier studies, some reported structures may require reevaluation. Importantly, the biflavonoids identified in these plants are often concentrated in non-edible parts, such as ginkgo leaves [19], mangosteen pericarps [18], and the outer layers of onions [20].

Beyond dietary sources, biflavonoids are commonly reported in medicinal plants used in both traditional and modern medicine, where they are often associated with notable pharmacological effects, including antimicrobial activity. Thus, they may also contribute to the antimicrobial activity. A selection of widely recognized medicinal plants with reported antimicrobial uses and their associated biflavonoids is summarized in Table 1.

### 2.2. Synthesis

Biflavonoids possess significant medicinal value and represent a promising class of compounds for future therapeutic development. Their diverse pharmacological activities suggest that demand for these molecules will increase, both for drug discovery and for fundamental research. However, the extraction and purification of biflavonoids from natural sources is challenging, often yielding only small quantities of material with limited purity. As a result, synthetic approaches are crucial for accessing sufficient amounts of biflavonoids for detailed biological evaluation [23].

Biflavonoids are dimeric structures derived from C4 carbonyl flavonoids such as chalcones, flavanones, flavones, flavanols, flavonols, aurones, and isoflavones. Their structural diversity arises from differences in the oxygenation patterns of the monomer units, the oxidation state at C3, and the nature of the interflavonoid linkage [24]. The interflavonoid bonds can form via the A-ring (positions 5, 6, 7, or 8), the B-ring (positions 2′, 3′, 4′, 5′, or 6′), or the C-ring (positions 2 or 3), leading to either C–C or C–O–C linkages. Accordingly, biflavonoids can be classified into structural categories such as AA, BB, AB, CC, and so forth, depending on which rings participate in bonding. This combinatorial flexibility, together with the variety of monomeric units, creates the potential for a vast chemical space. Indeed, nearly 600 biflavonoid structures have already been described [10], but many more remain undiscovered in nature, unsynthesized in the laboratory, or untested for biological activity. This represents a largely untapped library of structurally and pharmacologically valuable molecules.

To access this diversity, a range of synthetic strategies has been developed [25,26]. Each method presents unique advantages and limitations in terms of selectivity, yield, scalability, and environmental impact:

- Ullmann coupling forms C–C bonds through copper-catalyzed reactions in polar aprotic solvents at high temperatures [26]. While effective, the reaction often requires harsh conditions and provides only moderate yields [26].
- Electrophilic aromatic substitution exploits the reactivity of electron-rich flavonoid rings towards electrophiles. It can enable regioselective bond formation, though controlling product distribution can be difficult [27].
- Oxidative coupling, either enzymatic or chemical, is a versatile route to both C–C and C–O–C linkages [28]. Enzyme-catalyzed approaches, such as peroxidase-mediated coupling, proceed under mild, environmentally benign conditions. In contrast, chemical oxidants (e.g., potassium ferricyanide) provide flexibility but often generate complex product mixtures [23,29].
- Dehydrative coupling involves the condensation of flavonoid hydroxyl groups into ether linkages. This method is conceptually simple but requires careful catalyst choice to avoid degradation of sensitive functional groups.
- Modern cross-coupling reactions, particularly palladium-catalyzed methods such as Suzuki–Miyaura coupling [30,31,32], enable highly selective C–C bond formation. These strategies offer excellent control and efficiency but may be limited by the cost of catalysts and the need for precise reaction conditions.

Comparing the chemical and biochemical strategies, chemical strategies/approaches typically involve high temperatures, strong oxidizing agents, and multistep reaction schemes, resulting in low-to-moderate yields and limited practical applicability In contrast, biosynthetic approaches have emerged as promising and sustainable strategies, offering high efficiency and selectivity under mild and cost-effective conditions [10,33,34]. As research on the synthesis of natural flavonoid dimers has advanced, a variety of enzymes involved in flavonoid dimerization via biotransformation have been identified in both plants and microbiomes, including polyphenol oxidases [35], laccases [36], and cytochrome P450 monooxygenases [23,37]. Although biflavonoids were first identified nearly a century ago, beginning with the isolation of compound ginkgetin from Ginkgo biloba, the enzymes responsible for flavonoid dimerization remain unidentified, which continues to hinder progress in their biosynthetic development.

By tailoring both chemical and biosynthetic approaches to achieve specific structural features, researchers can systematically expand the repertoire of biflavonoids available for study. This synthetic accessibility not only allows for direct comparison between natural and synthetic dimers but also opens the door to the rational design of novel analogs with optimized pharmacological properties. Ultimately, the development of efficient synthetic strategies provides the foundation for unlocking the full therapeutic potential of biflavonoids.

## 3. Antimicrobial Activity of Biflavonoids

**Figure 2:**
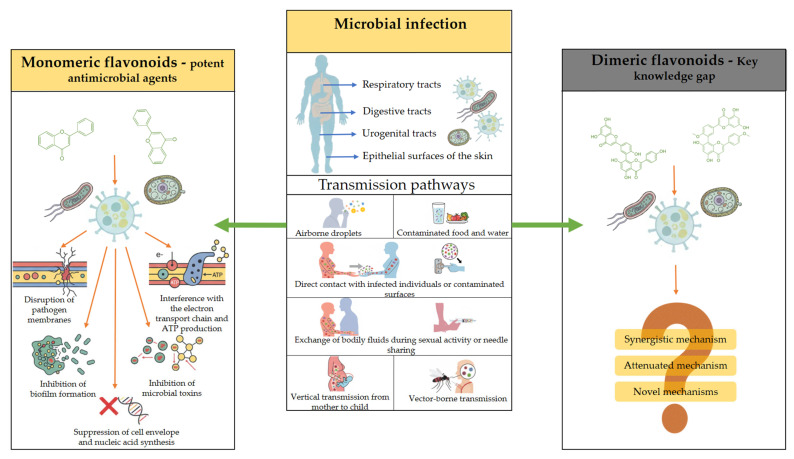
Schematic overview of microbial infections and antimicrobial mechanisms of flavonoids, highlighting the transition from well-studied monomeric flavonoids to underexplored biflavonoids.

Microbial infections can affect a wide range of hosts, including humans and animals, and may manifest in diverse ways. They commonly occur on epithelial surfaces of the skin and mucosa of the digestive, urogenital, and respiratory tracts [38]. Transmission pathways are equally diverse: airborne droplets, as in influenza, SARS, or the common cold; contaminated food and water, as in pathogenic *Escherichia coli* or hepatitis A; direct contact with infected individuals or contaminated surfaces, as in herpes simplex virus (HSV), certain bacteria, and fungi; exchange of bodily fluids during sexual activity or needle sharing; vertical transmission from mother to child, as in HIV, hepatitis B, and HSV; and vector-borne transmission, as in malaria and other viral infections [38] (Figure 2).

Once introduced into the host, microbes establish infection through mechanisms such as tissue penetration, adhesion, and colonization of epithelial surfaces. Progression to disease requires successful replication and dissemination within the host. These processes are counteracted by the immune system through inflammatory responses, phagocytosis, pathogen recognition, macrophage activation, and recruitment of natural killer cells, all of which act to limit or eliminate infection. Polyphenols, particularly flavonoids, have long been recognized as potent antimicrobial agents [39]. Numerous monomeric flavonoids have been characterized, and their antimicrobial effects often resemble the mechanisms of conventional drugs. Reported modes of action include disruption of pathogen membranes, inhibition of biofilm formation, suppression of cell envelope and nucleic acid synthesis, interference with the electron transport chain and ATP production, and inhibition of microbial toxins [39]. Importantly, the antimicrobial efficacy of flavonoids is highly structure-dependent, and several reviews have systematically examined the relationship between chemical modifications and biological activity [5,40].

However, a key knowledge gap remains: while the structural determinants of monomeric flavonoid activity are well studied, little is known about how dimerization influences antimicrobial properties. Understanding whether biflavonoids exhibit synergistic, attenuated, or novel mechanisms compared to their monomeric counterparts represents an important frontier in flavonoid research.

### 3.1. Antiviral Activity

Over the past two decades, biflavonoids have been the subject of extensive research as potential antiviral agents. These studies have revealed inhibitory activities against a variety of viruses, including respiratory viruses (such as influenza A, influenza B, respiratory syncytial virus, parainfluenza type 3, adenovirus type 5, measles, and severe acute respiratory syndrome, including SARS-CoV-2), herpes viruses (including HSV-1, HSV-2, human cytomegalovirus (HCMV), Varicella-zoster virus (VZV), and Epstein–Barr virus), human immunodeficiency virus (HIV), and viruses responsible for diseases such as dengue, coxsackievirus, and hepatitis [41].

**Figure 3:**
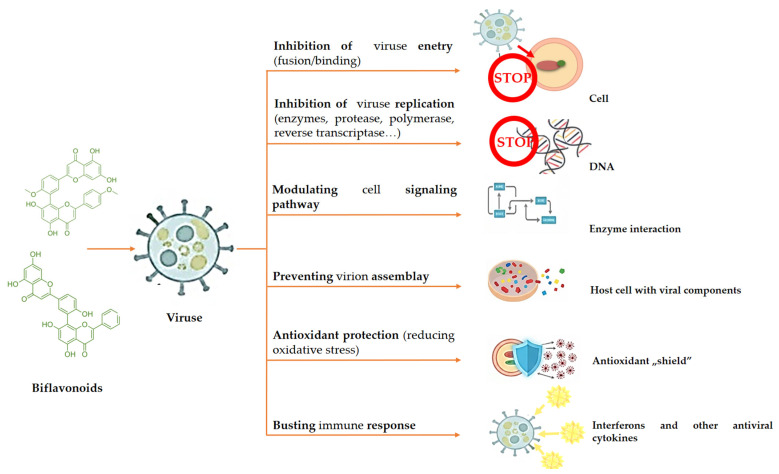
Schematic overview of the principal antiviral mechanisms attributed to biflavonoids. Biflavonoids may inhibit viral entry by blocking interactions between viral surface proteins and host cell receptors, interfere with viral replication through inhibition of viral enzymes such as polymerases and proteases, suppress viral assembly and release, and modulate host immune responses. Antioxidant and anti-inflammatory effects may further contribute to limiting viral propagation and host cell damage.

Biflavonoids have multiple scientifically proven activities that contribute to their antiviral mechanisms (Figure 3). By inhibiting the fusion of the viral envelope with the host cell membrane or obstructing the interaction between viral particles and host cell surface receptors, biflavonoids can hinder virus entry. Additionally, by targeting viral enzymes necessary for the production and processing of viral genetic material, such as polymerase, protease, and reverse transcriptase, they prevent viral replication. Moreover, biflavonoids can interfere with the assembly of viral components inside the host cell, stopping the development of mature virions. By influencing the production of interferons and other antiviral cytokines, they enhance the host’s immune response and strengthen the host’s defenses against viruses. Furthermore, biflavonoids possess antioxidant properties that reduce oxidative stress in host cells, making the environment less conducive to viral replication. These combined mechanisms facilitate the antiviral activity of biflavonoids against a variety of viruses, and there is extensive research on this antiviral mechanism in the literature [42].

In an effort to discover novel biflavonoid compounds that could combat the dengue fever virus (DV), Coulerie et al. [43] isolated four biflavonoids—amentoflavone, podovarpusflavone A, isoginkgetin, and hinokiflavone—from the *Dacrydium balansae* ethyl acetate extract. They discovered that these biflavonoids were the most potent inhibitors of the DV-NS5 RDRP and DV-NS5 (non-structural protein 5 of the dengue virus), with the half maximal inhibitory concentration (IC_50_) values below 3.1 and 5.3 μM. The most potent biflavonoid was hinokiflavone, with an IC_50_ of 0.26 μM. However, the most potent non-cytotoxic DV-NS5 inhibitor was podocarpusflavone A, which also had the ability to inhibit polymerase activity in the DV replicon. As a result, according to the authors, podocarpusflavone A can be used as a model for the development of medications that combat the DV [43].

Throughout the COVID-19 crisis, biflavonoids attracted significant scientific attention as natural compounds capable of targeting viral replication pathways. Most research to date has focused on amentoflavone, as its antiviral activity and potential mechanisms of action have been extensively investigated through both in vitro assays and in silico modeling, and thoroughly discussed in the literature research [44,45]. However, despite these promising findings, its efficacy and safety have not yet been validated in vivo, leaving an important gap in our understanding of its therapeutic potential. Biflavonoids sciadopitysin, ginkgetin, isoginkgetin, amentoflavone, and bilobetin have been comparably evaluated in combined in vitro and in silico studies as inhibitors of the SARS-CoV-2 3CLpro enzyme, which is essential for viral replication. Inhibition experiments demonstrated significant anti-SARS-CoV-2 activity, with sciadopitysin identified as the most effective inhibitor. Molecular docking and kinetic analyses further supported its reversible and heterogeneous inhibitory mechanisms [46]. Two naturally occurring anti-HIV biflavones, hinokiflavone and robustaflavone, were shown to inhibit the membrane fusion mechanism of the SARS-CoV-2 spike protein with human ACE2 receptors [47]. Similarly, a study by Lokhande et al. [48] using molecular docking and simulations identified six bioflavonoids from *Rhus succedanea*—amentoflavone, agathisflavone, robustaflavone, hinokiflavone, rhusflavanone, and succedaneaflavanone—as strong binders of the SARS-CoV-2 main protease (Mpro). These compounds interacted with the Mpro catalytic site (~19.47 to ~27.04 kcal/mol), with amentoflavone (~27.04 kcal/mol) and agathisflavone (~25.87 kcal/mol) showing the strongest interactions with catalytic residues [48]. Ghosh et al. [49] also evaluated four biflavonoids against Mpro, determining that amentoflavone, bilobetin, and ginkgetin possess favorable pharmacokinetic properties for human use. Abdizadeh et al. [50] investigated apigenin-based biflavonoid derivatives and found that these compounds form strong hydrophilic and hydrophobic interactions with one or both catalytic residues (His41 and Cys145) of 3CLpro. MD simulations confirmed that amentoflavone, bilobetin, ginkgetin, and sotetsuflavone form stable complexes with binding free energies of 59.36–65.84 kJ/mol, supported by DFT descriptors, pharmacokinetics, and ADMET profiles [50]. Agathisflavone was also identified as a potential anti-SARS-CoV-2 agent. In silico and enzymatic assays revealed that it non-competitively inhibits PLpro, but not Mpro. In Calu-3 cells, agathisflavone showed an EC_50_ of 4.23 ± 0.21 μM and a CC_50_ of 61.3 ± 0.1 μM, outperforming its monomer apigenin, and additionally suppressing TNF-α [51].

Except for SARS-CoV-2, amentoflavone has frequently been investigated for its antiviral activity against other clinically relevant viruses. Ma et al. [52] reported its inhibitory effect against RSV, a major cause of respiratory infections, demonstrating an IC_50_ value of 5.5 mg/mL in vitro. In addition, agathisflavone has shown potential against the influenza virus [53], and amentoflavone has been considered as a candidate for developing antiviral therapies against HSV-1 [54]. Amentoflavone’s ability to inhibit viral growth in human Raji cells infected with CVB3 was explored by Wilsky et al. [55], who found that treatment with amentoflavone and orlistat up to eight hours post-infection significantly reduced CVB3 replication and suppressed virus-induced FAS expression. These findings are consistent with Yin et al. [56], who investigated amentoflavone, the main constituent of *Selaginella moellendorffii*, and its total flavonoid extracts against CVB3. Both amentoflavone and the extracts inhibited cytopathic effects during or after infection, with IC_50_ values of 25 ± 1.2 to 52 ± 0.8 μg/mL for amentoflavone and 19 ± 1.6 to 41 ± 1.2 μg/mL for the extracts in MTT assays. Furthermore, Lee et al. [57] demonstrated that amentoflavone could inhibit resistant-associated variants of the NS5A inhibitor daclatasvir and interfere with multiple stages of the HCV life cycle, including viral entry, replication, and translation.

Taken together, these studies suggest that biflavonoids, especially amentoflavone, and their derivatives may have broad-spectrum antiviral potential against respiratory, enteric, and other clinically relevant viruses (Table 2), although further in vivo studies are required to confirm their efficacy and clarify their mechanisms of action.

**Table 2:** Studies related to the antiviral activity of biflavonoids.

Types of Biflavonoids	Source	Pathogen	Outcomes	References
Sciadopitysin Ginkgetin Isoginkgetin Amentoflavone Bilobetin	*Ginkgo biloba*	SARS-CoV-2	Bioflavones displayed relatively strong SARS-CoV-2 3CL^pro^ inhibition activities (IC_50_ < 10 μM).	[46]
Amentoflavone Bilobetin Ginkgetin Sciadopitysin	*Torreya nucifera*	SARS-CoV-2	Amentoflavone exhibited a significant antiviral activity (IC_50_ = 8.3 ± 1.2 μM). IC_50_ values of bilobetin, ginkgetin, and sciadopitysin were found as 72.3 ± 4.5 μM, 32.0 ± 1.7 μM, and 38.4 ± 0.2 μM, respectively.	[58]
Hinokiflavone Robustaflavone	-	SARS-CoV-2	The substances exhibited an inhibitory effect against the membrane-fusion-based SARS-CoV-2 spike protein invasion of target cells.	[47]
Amentoflavone Agathisflavone Robustaflavone Hinokiflavone Rhusflavanone Succedaneaflavanone	*Rhus succedanea*	SARS-CoV-2	Strong interactions are observed between Amentoflavone (~27.04 kcal/mol) and Agathisflavone (~25.87 kcal/mol) and the catalytic residues. All six biflavonoids bind with strong affinity to the same catalytic site of M^pro^, as demonstrated by molecular interactions and molecular dynamics.	[48]
Amentoflavone Bilobetin Ginkgetin	*Torreya nucifera*	SARS-CoV-2	Of the four phytochemicals, only three (amentoflavone, *k*_i_ = 0.17 mM; bilobetin, *k*_i_ = 0.21 mM; and ginkgetin, *k*_i_ = 0.26 mM) exhibited higher binding affinities than lopinavir and N3.	[49]
Amentoflavone Bilobetin Ginkgetin Sotetsuflavone	-	SARS-CoV-2	Significant hydrophilic and hydrophobic bonding interactions were observed between the biflavonoid compounds and either or both catalytic residues (His41 and Cys145) of 3CLpro.	[50]
Agathisflavone	*Anacardium occidentale*	SARS-CoV-2	Agathisflavone therapy of SARS-CoV-2 replication is effective, with EC_50_ and CC_50_ values of 4.23 ± 0.21 and 61.3 ± 0.1 μM, respectively.	[51]
Amentoflavone Podovarpusflavone A Isoginkgetin Hinokiflavone	*Dacrydium balansae*	DV	Biflavonoids were the most potent inhibitors of the DV-NS5 RDRP and DV-NS5, with IC_50_ values below 3.1 and 5.3 μM. The most potent biflavonoid was hinokiflavone, with an IC_50_ of 0.26 μM.	[43]
Amentoflavone	*Selaginella sinensis*	RSV	The IC_50_ value of amentoflavone was 5.5 mg/mL.	[52]
Amentoflavone	-	HSV	Amentoflavone exhibited considerable antiviral action against HSV-1 and significantly reduced transcription of viral immediate early genes.	[54]
Amentoflavone	-	CVB3	Amentoflavone and orlistat dramatically decreased CVB3 replication and suppressed virus-induced FAS expression.	[55]
Amentoflavone	*Selaginella moellendorffii*	CVB3	The total extracts’ IC_50_ values in the MTT experiment ranged from 19 ± 1.6 to 41 ± 1.2 μg/mL, whereas amentoflavone’s values ranged from 25 ± 1.2 to 52 ± 0.8 μg/mL.	[56]
Amentoflavone	-	HCV	It had inhibitory effects on resistant-associated variants of the NS5A inhibitor daclatasvir, as well as on viral entry, replication, and translation of the HCV life cycle.	[57]
Agathisflavone	*Anacardium occidentale* L.	Influenza	The influenza virus was suppressed by agathisflavone, with an EC_50_ of 1.3 μM.	[53]

### 3.2. Antibacterial Activity

**Figure 4:**
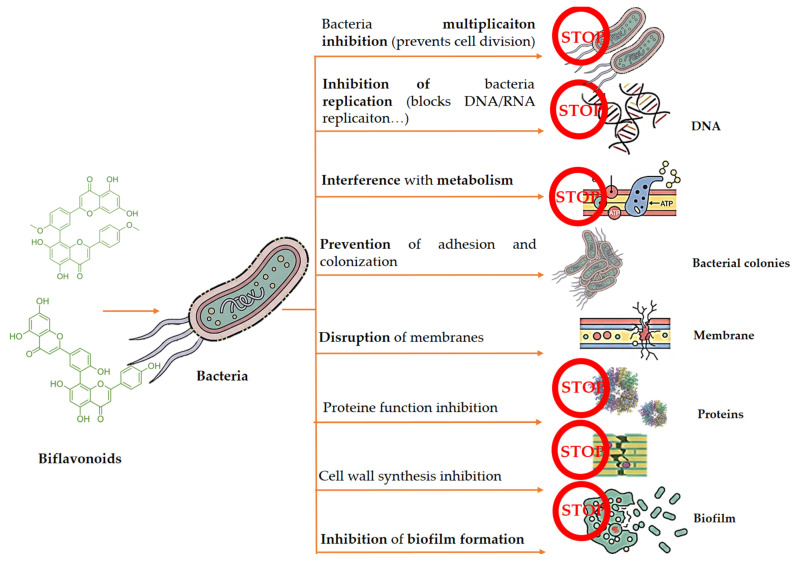
Schematic overview of the antibacterial actions of biflavonoids against Gram-positive and Gram-negative bacteria. Proposed mechanisms include disruption of bacterial cell membranes, inhibition of cell wall and nucleic acid synthesis, suppression of biofilm formation, interference with energy metabolism, neutralization of bacterial toxins, and modulation of virulence factors. Dimerization of flavonoids may enhance membrane interactions and target binding compared with monomeric flavonoids.

Biflavonoids play a role in responding to microbial infections [41]. Considering the literature, studies suggested that biflavonoids have considerable antibacterial activity, which could assist in delaying the progression of various pathogens. While the specific antibacterial mechanisms of biflavonoids are yet to be fully elucidated, it is currently hypothesized that they rely on the known actions of their monomeric flavonoid counterparts [59]. Summarized information is presented in Table 3 and Figure 4.

Six biflavonoids were identified from *Ochna macrocalyx* bark by Tang et al. [68]. In addition to ochnone, cordigol, calodenin B, and 2,3-dihydrocalodenin B being distinct from ordinary biflavonoids, the C-C connected biflavonoids are dehydroxyhexaspermone C and hexaspermone C. While demonstrating high antibacterial properties, both calodenin B and 2,3-dihydrocalodenin B exhibit some cytotoxicity. The antibacterial properties of 2,3-dihydrocalodenin B and calodenin B were more evident when compared to the control medication [68]. Furthermore, fukugiside has the ability to stop *Streptococcus pyogenes* from acting [69]. Amentoflavone was tested by Hwang et al. [60] for its antimicrobial activity against a variety of bacterial strains, including *Enterococcus faecium*, Staphylococcus aureus, *Streptococcus mutans*, *E. coli* O-157, and *Pseudomonas aeruginosa*. The findings demonstrated the exceptional antibacterial activity of amentoflavone against both Gram-positive and Gram-negative bacteria, with minimum inhibitory concentration (MIC) values ranging from 4 to 32 µg/mL. Amentoflavone and all the antibiotics were less effective against *S. mutans* than other bacterial strains, according to the results of the synergistic interaction. Cefotaxime greatly reduced the sensitivity of the Gram-positive bacteria *E. faecium* and *E. coli* O-157 and *P. aeruginosa. P. aeruginosa*, *E. coli* O-157, and *E. faecium* all showed equal MIC values for amentoflavone. Also, amentoflavone exhibited strong antibacterial activity that was comparable to that of the antibiotics (ampicillin, cefotaxime, and chloramphenicol). Thus, the results demonstrated that amentoflavone had a significant antibacterial effect and synergistic interaction when combined with antibiotics [60]. Amentoflavone has demonstrated antimicrobial activity against foodborne pathogens such as *S. aureus* and *E. coli*, as verified in food models using minced chicken meat and apple juice [61].

*Streptococcus* pneumoniae’s pneumolysin (PLY) is a toxic substance that ruptures cell membranes, disrupting cells and inflaming them. Amentoflavone can prevent PLY oligomerization by interacting with the Ser254, Glu277, and Arg359 sites, as demonstrated by Zhao et al. [62]. This prevents PLY-mediated damage to human alveolar epithelial cells [62]. A major zoonotic infection, *Streptococcus suis*, can cause significant financial losses for the swine industry [70]. It produces suilysin (SLY), an extracellular toxin that forms pores and can cause necrosis, apoptosis, and cell lysis in a range of host cells [71]. Amentoflavone reduces the cytotoxicity that *S. suis* induces in macrophages and effectively inhibits SLY oligomerization. Additionally, amentoflavone reduced inflammation in S. suis-infected cells by regulating the p38, JNK1/2, and NF-κB pathways [63].

Research on *Rhus natalensis* has resulted in the identification of a new biflavonoid with antibacterial characteristics named rhuschromone by Mwangi et al. [64]. It was discovered that rhuschromone had a comparatively high activity against *S. aureus* ATCC 25923, which was equivalent to the standard of chloramphenicol. Nevertheless, more thorough research would be needed to determine whether rhuschromone would be a good option for the creation of innovative drugs. Negm et al. [66] isolated one new biflavonoid, 5,7,7″,4‴-tetra-O-methyl-hinokiflavone, and five recognized compounds—sigmasterol, naringenin, 2,3-dihydrobilobetin, 4′,4‴-O-dimethyl amentoflavone, and hinokiflavone from the *Cycas thouarsii* R.Br. leaves extract. The MICs of the pure compounds ranged from 0.25 to 2 µg/mL against clinical isolates of *Klebsiella pneumoniae*. Five biflavonoids, including two new ones, were extracted from the aerial portions of *Ormocarpum trichocarpum* using activity-guided fractionation based on in vitro antibacterial testing [72]. The MIC values of these compounds against *S. aureus*, *Bacillus subtilis*, *E. coli*, and *K. pneumoniae* ranged from 4.0 to 136.7 µM, whereas the IC_50_ values against the chloroquine-sensitive D10 *Plasmodium falciparum* strain revealed a range of 4.30 to 94.32 µM. This example demonstrates that biflavonoids are frequently isolated as active compounds from plant extracts exhibiting antibacterial activity, suggesting their potential use as antibacterial agents. However, compared with monomeric flavonoids, research on biflavonoids remains relatively limited. Further studies are therefore necessary to elucidate how dimerization influences antibacterial properties and to what extent structural features of biflavonoids contribute to their biological activity.

### 3.3. Antifungal Activity

**Figure 5:**
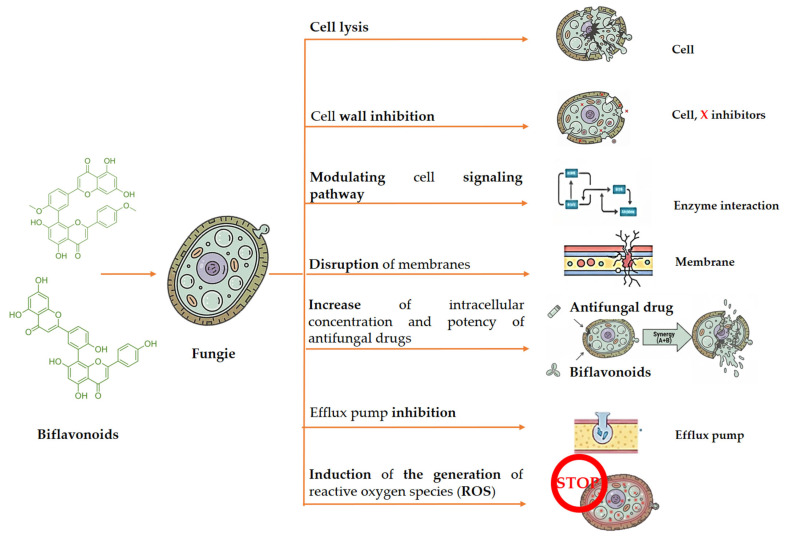
Schematic overview of the main antifungal mechanisms associated with biflavonoids. These include disruption of fungal cell membrane integrity, inhibition of cell wall biosynthesis, induction of oxidative stress through reactive oxygen species (ROS) generation, interference with intracellular signaling pathways, and inhibition of efflux pumps. Collectively, these effects can impair fungal growth, morphology, and viability.

Biflavonoids have several mechanisms underlying their antifungal action (Figure 5). They can disrupt fungal cell membranes, leading to cell lysis and death. By impeding the synthesis of essential enzymes for cell walls, such as chitin synthase and β-glucan synthase, they hinder cell wall formation. Additionally, biflavonoids induce the generation of reactive oxygen species (ROS) in fungal cells, causing oxidative damage to proteins, lipids, and DNA. They can also obstruct fungal cell signaling pathways, interfering with processes related to growth, division, and differentiation. Moreover, biflavonoids may inhibit efflux pumps, increasing the intracellular concentration and potency of antifungal drugs. These combined effects make biflavonoids effective against a variety of fungal infections [10].

Summarized information about biflavonoid antifungal activity is presented in Table 4.

*Candida albicans* is the primary model organism used to evaluate the antifungal activity of biflavonoids. To investigate the mechanism of action of the biflavonoid isocryptomerin, Lee et al. [78] employed bis-(1,3-dibutylbarbituric acid) trimethine oxonol [DiBAC_4_(3)], a conventional membrane potential dye, in a regeneration assay with fungal protoplasts. The study demonstrated the antifungal potential of isocryptomerin against human pathogenic fungi, including *C. albicans* and *Trypanosoma beigelii*, with a minimum inhibitory concentration (MIC) of 18.11 μM, using amphotericin B as a positive control. Interestingly, DiBAC_4_(3) uptake induced by isocryptomerin was negligible and significantly lower than that observed with amphotericin B, indicating that its antifungal effect primarily results from disruption of plasma membrane integrity rather than membrane depolarization [78].

Ochnaflavone, another biflavonoid, has been shown to modulate inflammatory responses in *C. albicans*-induced fungal arthritis [79]. It inhibited the expression of pro-inflammatory cytokines such as IFN-γ and IL-2, while upregulating anti-inflammatory cytokines IL-4 and IL-10 via T-cell mediated pathways. However, ochnaflavone did not induce hemolysis, eliminate excess macrophages, or fully ameliorate fungal arthritis symptoms [79]. Amentoflavone, isolated from *Selaginella tamariscina*, has demonstrated significant antifungal activity against several pathogenic fungi, including *C. albicans*, *Saccharomyces cerevisiae*, and *Trichosporon beigelii*. This compound was also evaluated for its effects on *C. albicans* dimorphism and cytotoxicity through hemolytic assays on human erythrocytes. The results showed minimal hemolytic activity while exhibiting strong antifungal efficacy at concentrations of 5–10 μg/mL, highlighting its potential as a therapeutic antifungal agent [73]. Further studies have confirmed the potent antifungal activity of amentoflavone against *C. albicans*. Its mechanism of action appears to involve physiological alterations leading to S-phase arrest within the fungal cells [60]. Hwang et al. [75] also demonstrated that amentoflavone induces mitochondrial dysfunction and triggers apoptotic cell death in *C. albicans*.

Similarly, the antifungal properties of biflavonoids such as amentoflavone, bilobetin, sequoiaflavone, ginkgetin, sciadopitysin, and 2,3-dihydrosciadopitysin were assessed using computer-aided image analysis microscopy. Derived from *Taxus baccata* and *Ginkgo biloba*, these compounds were tested against *Alternaria alternata*, *Fusarium culmorum*, and *Cladosporium oxysporum*. At 100 μM, bilobetin completely inhibited the growth of *F. culmorum* and *C. oxysporum*, with effective dose (ED_50_) values of 14, 11, and 17 μM, respectively. While bilobetin was less effective than ginkgetin and 7-O-methylamentoflavone against *A. alternata*, exposure to 200 μM ginkgetin caused pronounced structural alterations in the fungal cell wall [76]. In another investigation, the concentration-dependent antifungal effects of biflavonoids—including amentoflavone, bilobetin, ginkgetin, isoginkgetin, and sciadopitysin—were compared to their monomeric flavonoid counterparts, such as apigenin, genkwanin, and acacetin [77]. All biflavonoids showed strong inhibition of *Fusarium graminearum* at 0.01 μg/mL, outperforming the monomeric compounds. Against *Aspergillus ochraceus*, biflavonoids and genkwanin were more active at lower concentrations, although their activity decreased at higher doses. Conversely, *A. flavus* was inhibited in a concentration-dependent manner by biflavonoids, while apigenin and genkwanin had no effect on *A. alternata*. These findings suggest that dimerization may enhance antifungal activity, although further investigation is needed to fully understand this effect.

## 4. Conclusions

In this review, we have summarized current knowledge on the occurrence, synthesis, and antimicrobial activity of biflavonoids. Most of the available data focus on the biflavonoid amentoflavone, a dimer of apigenin [3′–8′]. This emphasis is likely due to its widespread natural occurrence and the commercial availability of a standard compound. The available evidence indicates that biflavonoids exhibit distinct biological properties compared to their monomeric flavonoid counterparts, suggesting that dimerization significantly influences their antimicrobial potential. However, the exact relationship between structural features, such as the type and degree of linkage, and biological activity remains to be fully elucidated.

Particularly noteworthy is the promising antiviral potential of biflavonoids. Numerous in silico and in vitro studies have highlighted their ability to interact with viral proteins and inhibit viral replication, positioning them as attractive candidates for antiviral drug development. Nevertheless, comprehensive in vivo investigations are still required to validate these findings, establish effective concentrations, determine pharmacokinetic parameters, and evaluate safety profiles.

Future research should prioritize structure–activity relationship (SAR) studies, advanced synthetic approaches to generate diverse biflavonoid derivatives, and the investigation of their potential synergistic effects with existing antimicrobial and antiviral agents. Equally important is the need to conduct comprehensive in vivo experiments to validate the efficacy and safety of these compounds in biological systems. Detailed mechanistic studies are also essential to understand how biflavonoids exert their antimicrobial and antiviral effects at the molecular and cellular levels. Together, these efforts will provide critical insights into their pharmacological potential and may pave the way for the development of novel biflavonoid-based therapeutics with broad-spectrum activity and clinical relevance.

## Figures and Tables

**Table 1:** Examples of plant species accumulating biflavonoids, with reported compounds and their biflavonoid types.

Latin Name	Biflavonoids-Type	Biflavonoids
*Hypericum perforatum* L.	3′,8″ 3,8″	amentoflavone 3,8″-biapigenin [21]
*Selaginella* sp.	Seven dimeric linking types: 2′-8″, 3-3″,3′-6″,3′-8″,3-O-4‴, 3′-O-4‴ and 4′-O-6′	2′,8′-biapigenin, taiwaniaflavone, 7,4′-di-O-methylrobus taflavone, 7″-O-methylrobustaflavone, 4′-O-methylrobustaflavone, robustaflavone, 7,4′-di-O-methyl-2″,3″-dihydrorobustaflavone, 7, 4′,7″-tri-O-methyl-2″,3″-dihydrorobustaflavone, 7,4′,7″,4‴-tetra-O-methylamentoflavone, kayaflavone, heveaflavone, ginkgetin, 7,7″-di-O-methylamentoflavone, 4′,7″-di-O-methylamento flavone, isoginkgetin, bilobetin, podocarpusflavone A, sostetsuflavone, amentoflavone, sumaflavone, 2,3-dihy droamentoflavone, 2″,3″-dihydroamentoflavone, tetrahy droamentoflavone, delicaflavone, ochnaflavone, crypto merin B, pulvinatabiflavone, 7-O-methyl-hinokiflavone, isocryptomerin, hinokiflavone, 2″,3″-dihydroisocryptomerin, 2,3-dihydrohinokiflavone (45), 2″,3″-dihydrohinokiflavone, tetrahydrohinokiflavone [22]
*Juniperus* sp.	3′,8″, 8,8″, 4′,6″	amentoflavone, bilobetin, cupressuflavone, hinokiflavone [15]
*Ginko biloba* L.	3′,8″	ginkgetin, isoginkgetin, amentoflavone, bilobetin, sciadopitysin, sesquoiaflavone, podocarpusflavone A, and 5′-metoxybilobetin [19]
*Garcinia madruno* L.	3′,8″ 3″-8″	amentoflavone, morelloflavone, volkensiflavone, fukugiside, spicataside [17]

**Table 3:** Studies related to the antibacterial activity of biflavonoids.

Types of Biflavonoids	Source	Pathogen	Outcomes	References
Amentoflavone		*E. faecium* *S. aureus* *S. mutans* *E. coli* O-157 *P. aeruginosa*	Amentoflavone showed remarkable antibacterial activity against Gram-positive and Gram-negative bacteria, with MIC values of 4–32 µg/mL.	[60]
Amentoflavone	*Nandina domestica*	*S. aureus* KCTC 1621 *E. coli* ATCC 43889	*S. aureus* and *E. coli* showed a significant reduction in cell viability at their respective MICs of 62.5 and 125 µg/mL.	[61]
Amentoflavone	-	*S. pneumoniae*	By interacting with the toxin at Ser254, Glu277, and Arg359, amentoflavone efficiently inhibits the oligomerization of wild-type PLY and shields human alveolar epithelial (A549) cells from harm caused by pneumolysin.	[62]
Amentoflavone	Chinese herbs	*S. suis*	Although amentoflavone does not significantly affect the expression of SLY, it was found to be a strong antagonist of SLY-mediated hemolysis.	[63]
Rhuschromone	*Rhus natalensis*	*S. aureus* *E. coli* *P. aureginosa*	Rhuschromone had a comparatively high activity against *S. aureus*.	[64]
GB1	*Garcinia kola Heckel*	*S. mutans*	GB1 showed activity against *S. mutans* and other oral bacteria with MIC values of 32–64 µg/mL.	[65]
5,7,7″,4‴-tetra-O-methyl-hinokiflavone 2,3-dihydrobilobetin 4′,4‴-O-dimethyl amentoflavone Hinokiflavone	*Cycas thouarsii* R.Br.	*K. pneumoniae*	The MICs of the pure compounds ranged from 0.25 to 2 µg/mL against pathogens.	[66]
Ericoside	*Erica mannii*	*E. coli* AG100	With a MIC of 64 μg/mL, ericoside showed moderate efficacy against the resistant bacteria.	[67]

**Table 4:** Studies related to the antifungal activity of biflavonoids.

Types of Biflavonoids	Source	Pathogen	Outcomes	References
Amentoflavone	*Selaginella tamariscina*	*C. albicans* *S. cerevisiae* *T. beigelli*	Amentoflavone exhibited a significant antifungal activity at concentrations ranging from 5–10 µg/mL.	[73]
Amentoflavone	*Selaginella tamariscina*	*C. albicans*	Cell cycles were markedly inhibited during the S-phase by amentoflavone.	[74]
Amentoflavone	*Selaginella tamariscina*	*C. albicans*	Amentoflavone causes *C. albicans* cells to undergo apoptosis and is linked to mitochondrial malfunction.	[75]
Amentoflavone Bilobetin Sequoiaflavone Ginkgetin Sciadopitysin 2,3-Dihydrosciadopitysin	*Taxus baccata* *Ginkgo biloba*	*A. alternata Cladosporium oxysporum* *F. culmorum*	Bilobetin exhibited a significant antifungal activity with values of ED_50_ 14, 11, and 17 μm, respectively. Bilobetin was less effective than ginkgetin and 7-O-methylamentoflavone in their interactions with *A. alternata*. After being exposed to a 200 μm concentration of ginkgetin, *A. alternata* showed modest structural alterations in its cell wall.	[76]
Amentoflavone Bilobetin Ginkgetin Isoginkgetin Sciadopitysin	*-*	*A. alternata* *A. flavus* *A. ochraceus* *F. graminearum* *Fusarium verticillioides*	The fungus species and measured concentration had a significant impact on the antifungal activity. All the substances showed considerable inhibition of *F. graminearum* at 0.01 μg/mL, with biflavonoids working better than monomers.	[77]
Isocryptomerin	*Selaginella tamariscina*	*C. albicans* *T. beigelii* *S. cerevisiae*	Isocryptomerin, with an MIC value of 18.11 μM, exhibited against human pathogenic fungi.	[78]

## Data Availability

No new data were created or analyzed in this study. Data sharing is not applicable to this article.
